# Identification of osteoarthritis-related genes and potential drugs based on single cell RNA-seq data

**DOI:** 10.1186/s10020-025-01379-z

**Published:** 2025-11-25

**Authors:** Ning Wang, Kun Liu, Jia-Li Li, Wei-Wei Pang, Fu-Rong Zhang, Qin Zeng, Yun Deng, Xiao-Chao Qu, Xiang-Ding Chen, Hong-Wen Deng, Li-Jun Tan

**Affiliations:** 1 Laboratory of Molecular and Statistical Genetics, College of Life Sciences, Hunan Normal University, Changsha, 410081 Hunan China; 2 Zebrafish Genetics Laboratory, College of Life Sciences, Hunan Normal University, Changsha, 410081 China; 3 Tulane Center of Biomedical Informatics and Genomics, Deming Department of Medicine, Tulane University School of Medicine, 1440 Canal St., Suite 1610, New Orleans, LA 70112 USA

**Keywords:** Osteoarthritis, Mendelian randomization, Drug repositioning, Network-based analyses

## Abstract

Osteoarthritis (OA) is a global problem that seriously affects human health. At present, there is still a lack of effective drugs to treat OA. Therefore, we need to find more drugs with preventive and therapeutic effects on OA. In this study, we obtained single-cell RNA sequencing (scRNA-seq) and bulk-RNA seq datasets from Gene Expression Omnibus (GEO). By using high-dimensional weighted correlation network analysis (hdWGCNA), random forest method and protein–protein interaction (PPI) network analyses, five key genes (CXCL8, CCL20, MMP3, BIRC3 and ICAM1) related to OA were identified and the RT-qPCR experiments verified the differential expression of CXCL8, CCL20 and BIRC3 between Triclocarban (TCC) treated zebrafishes and controls. The SAVERUNNER algorithm predicted 42 candidate drugs. Mendelian randomization (MR) of the candidate drugs showed that the increased expression of TUBB1 led to a reduced risk of OA (β = -0.08, *P*-value = 4.56E-04), while Cabazitaxel (a microtubule dynamics inhibitor commonly used in the treatment of advanced prostate cancer) inhibits the expression of TUBB1, thus increases the risk of OA. Pitavastatin (a statin lipid-lowering drug that can reduce blood lipid levels and the risk of cardiovascular diseases) target genes expression (for HMGCR $\upbeta$= 0.13, *P*-value = 2.67E-06, for ITGAL $\upbeta$= 0.08, *P*-value = 6.57E-08) leads to an increased risk of OA, while Pitavastatin inhibits the expression of target genes, thus reduces risk of OA. The zebrafish experiments showed that Pitavastatin can increase the joint space of TCC treated OA zebrafish, while Cabazitaxel can decrease the joint space of TCC treated OA zebrafish. The RT-qPCR results of zebrafish verified that Pitavastatin inhibited the expression of HMGCR, while Cabazitaxel inhibited the expression of TUBB1. Our study suggested that Pitavastatin has therapeutic effects on OA, while Cabazitaxel increases the risk of OA.

**Supplementary Information:**

The online version contains supplementary material available at 10.1186/s10020-025-01379-z.

## Introduction

Osteoarthritis (OA) is the most common chronic joint disease in the world, characterized by cartilage degeneration, subchondral bone sclerosis, osteophyte formation and synovial joint inflammation (Martel-Pelletier et al. 2016). Currently with continuous prevalence of global obesity and an aging population, the prevalence of OA is gradually increasing and it can significantly alter joint dysfunction in the elderly, leading to disability and reduced quality of life. Previously the etiology of OA was usually thought to be mechanical strain leading to cartilage degradation. However, the current widely accepted hypothesis for the pathogenesis of OA suggests that OA development is driven by a complex interplay of factors, including genetic predisposition, inflammation, and changes in the joint microenvironment. While biomechanical stress may contribute to joint damage, it is not the sole or primary cause. Instead, initial damage to joint structures, regardless of its origin, triggers the release of inflammatory cytokines and activation of inflammatory pathways, which play a central role in the disease progression (Davidson et al. 2021). Reduction of cartilage tissue and narrowing of the joint space are hallmark pathophysiological mechanisms of OA (Martel-Pelletier et al. 2016). Zebrafish possess a well-characterized skeletal structure, making them an ideal model for studying joint and cartilage pathologies (Tonelli et al. 2020). Triclocarban (TCC, an antibacterial agent used in liquid soaps and body washes) has been shown to inhibit the expression of type II collagen and other extracellular matrix components, leading to joint space narrowing. As a result, the TCC-induced zebrafish model is widely used to study OA pathogenesis (Zhang et al. 2023).

In the development of OA, synovium show significant changes, mononuclear cell infiltration, thickening of the synovial lining layer, and production of inflammatory cytokines occur even before visible cartilage degeneration occurs (Mathiessen and Conaghan 2017). The prevalence of synovial inflammation is high in all stages of OA, and many studies have shown that synovial inflammation is associated with pain, poor function, and may even be an independent driver of OA episodes and structural progression (Mathiessen and Conaghan 2017). Therefore, the treatment of synovitis has great potential for pain relief and structural changes in OA.

Fibroblasts, the main cellular components resident in synovial tissue, have recently been thought to play a crucial role in the development and progression of OA (Manferdini et al. 2020). Fibroblasts in chronically infected, inflamed, and cancerous tissues become a key cell type in regulating the activation or suppression of immune responses. In normal human synovium, synovial fibroblasts occur as two subtypes, intimal fibroblasts and subintimal fibroblasts. Intimal fibroblasts are referred to as “fibroblast-like” or “type B” synoviocytes/synovial lining cells with phenotypic characteristics of macrophages and fibroblasts. These cells and the underlying vascularized connective tissue matrix form a complex structure that is an important source of synovial fluid components that are essential for normal cartilage and joint function (Scanzello and Goldring 2012). The role of fibroblasts varies according to their tissue of origin and the type of disease they induce, from maintaining a potent inflammatory milieu in chronic inflammation to promoting immunosuppression in malignant tumors and encapsulating and incarcerating infectious agents in tissues (Davidson et al. 2021). In mouse models of regressive and persistent arthritis, the absence of FAPα fibroblasts affects inflammation and bone erosion (Mathiessen and Conaghan 2017, Croft et al. 2019). Activated fibroblast-like synoviocytes (FLS) in osteoarthritic synovium secret cytokines, growth factors, matrix metalloproteinases (MMP) and tissue inhibitor of metalloproteinases (TIMP), which contribute to macrophage activation and stimulate catabolic pathways in chondrocytes (Sanchez-Lopez et al. 2022).

**Fig. 1:**
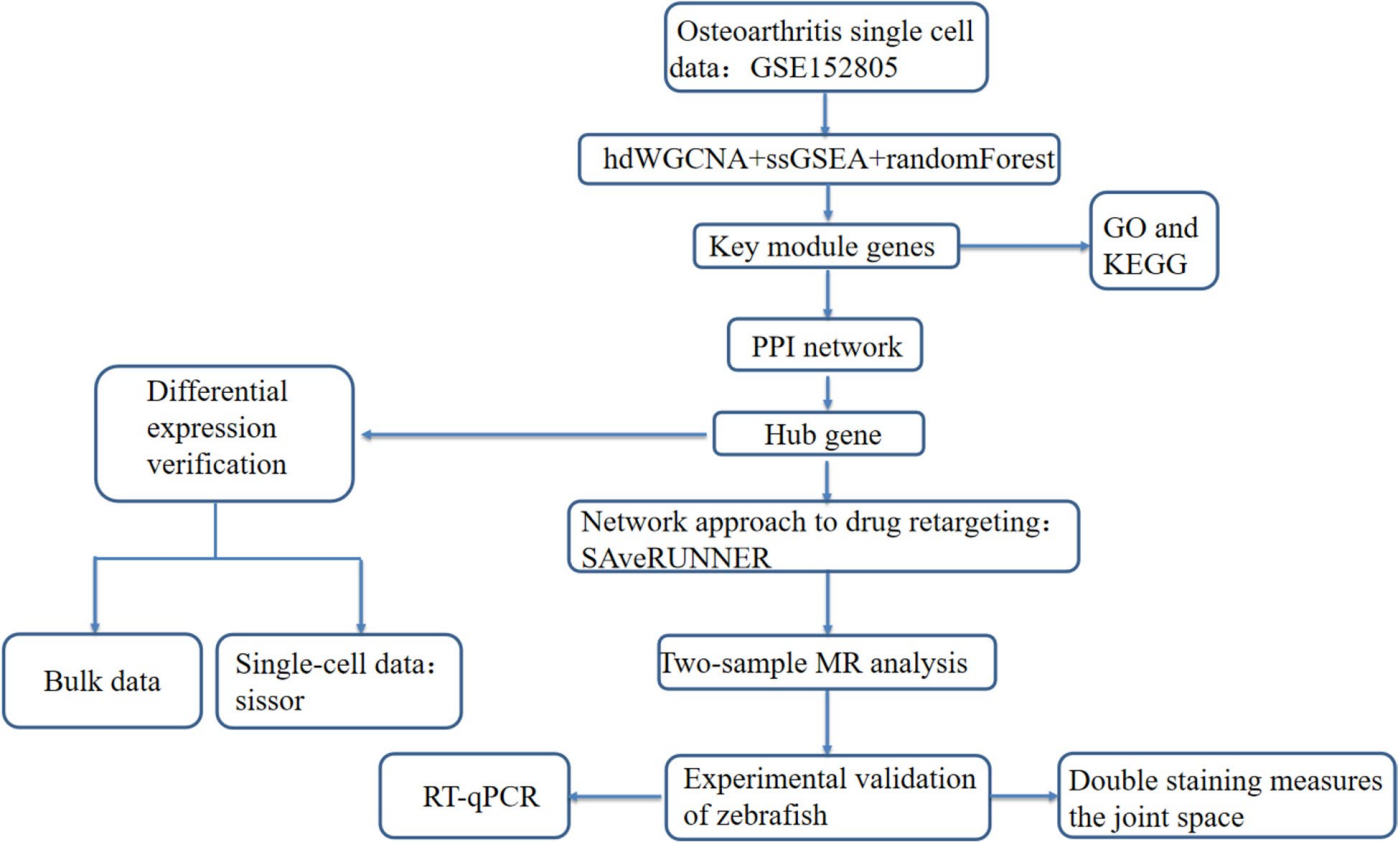
Schema of the study

In the current study, we performed bioinformatics analysis to determine the inflammation-related genes in fibroblasts of OA, and identified five inflammation-related key genes. Candidate drugs for OA treatment were screened by the SAVERUNNER algorithm based on the network proximity on the basis of the five genes identified above. Mendelian randomization (MR) analysis was performed to explore the causal relationship between candidate drugs’ target gene expression and OA. Finally, the zebrafish OA model was constructed using TCC, and the effect of the candidate drug on OA disease was verified by the measurement of the average joint gap. The five inflammation-related key genes and drug target genes were validated using RT-qPCR in zebrafish. The detailed schematic of the workflow in the current study is shown in Fig. 1.

## Materials and methods

### Obtainment of OA datasets

We downloaded three sequencing datasets of osteoarthritic synovial tissues from the Gene Expression Omnibus (GEO) database (https://www.Ncbi.nlm.nih.gov/geo/). Dataset GSE152805 (Chou et al. 2020) was based on the single-cell 10 × genomics sequencing platform, from which we selected three synovial tissue samples. Dataset GSE89408 (Guo et al. 2017) was based on the Illumina HiSeq 2000 RNA sequencing platform with 28 normal samples and 22 OA samples. Dataset GSE46750 (Lambert et al. 2014) was based on the Illumina HumanHT-12 V4.0 expression beadchip platform containing synovial tissue with inflammation or normal synovial tissue from 12 OA patients. The inflammation status of the normal and inflammatory synovial membrane in this dataset was characterized by the surgeon according to macroscopic criteria established by Ayral (Ayral 1996).

### Single-cell RNA-seq data analysis

We used Seurat (v4.3.0) for R environment (v.4.3.2) for scRNA-seq data analysis (Stuart et al. 2019). Quality control was implemented by filtering cells with > 10% mitochondrial genes and < 200 or > 7000 assay genes. To eliminate batch effects in the data from the three scRNA-seq samples, we used Harmony according to the official guidelines (Korsunsky et al. 2019). Then we performed uniform manifold approximation and projection (UMAP) via the RunUMAP function. Cell clusters were identified using the FindNeighbors and FindClusters (resolution = 0.1) functions. All the analysis procedures were performed using default parameters. The FindAllMarkers function of the Seurat was used to identify differentially expressed genes (DEGs) in each cluster using an adjusted *P-value* < 0.05 and |log FC|≥ 1 as statistical criteria. The cellular annotation of each cluster was performed using the marker gene information according to the previous study (Chou et al. 2020).

### Calculate inflammation score of cells

Since scRNA-seq dataset GSE152805 did not recruit healthy subjects of synovial tissue, we used inflammation status to represent healthy and diseased fibroblasts to identify inflammation-associated genes and gene modules. To calculate an inflammation score for each cell, single-sample gene set enrichment analysis (ssGSEA) were performed using the GSVA (Subramanian et al. 2005) package with a set of 200 genes associated with inflammatory response from MSigDB (Molecular Signature Database). The fibroblasts were classified into high inflammation and low inflammation groups based on the median of the inflammation scores.

### hdWGCNA analysis

To identify key gene modules associated with osteoarthritis, we performed high-dimensional weighted correlation network analysis (hdWGCNA) (Morabito et al. 2023) on fibroblast clusters of the single-cell dataset GSE152805, which is a new algorithm that provides a high degree of modularity to construct co-expression networks across multiple scales of cellular and spatial hierarchies and is more suitable for scRNA-seq data. Module Eigengenes (MEs) were defined as the first principal component of each gene module and the expression of MEs was considered as a representative of all genes in a given module. The eigengene-based connectivity of each gene, also known as kME was used to filter the highly connected genes within the module. A soft strength value of 9 was chosen to construct the co-expression network. The genes that cannot be assigned to any module constituted the gray module, so we did not analyze the gray module further.

### Identification of key modules associated with synovial inflammation

We used a random forest (RF) algorithm to identify key hdWGCNA modules on fibroblast clusters of the single-cell dataset GSE152805 for OA synovial inflammation. The MEs from hdWGCNA on the high/low inflammation groups of synovial fibroblasts were used as inputs for the random forest algorithm. RF analysis was performed using the R package randomForest, and the Gini coefficient (Gini Index) was used as the screening criterion. A higher value of the Gini coefficient indicates that the feature has a greater impact on the classification results, meaning that the module is more relevant to OA.

### PPI network analysis to identify inflammation-related key genes

In order to identify the inflammation-related key genes associated with OA synovitis, we utilized the PPI (protein–protein interaction) network approach for further analysis. Based on the connectivity of the genes, i.e., kME scores, the top 20 genes in the key module were selected to perform PPI analysis using Search Tool for Online Database Resources for Interacting Genes (STRING) (https://string-db.org/cgi/input.pl). The interactions of genes/proteins were visualized by Cytoscape (3.10.0) (Killcoyne et al. 2009). CytoHubba, a built-in plug-in for Cytoscape, can accurately filter out the critical nodes in the network with 11 methods of topology analysis based on the shortest path (Chin et al. 2014). We used the MCC (maximal clique centrality) algorithm of CytoHubba to obtain the key sub-network, and the genes in the key sub-network are used as key genes.

### The differential expression analysis of inflammation-related key genes

In order to verify whether the inflammation-related key genes are related to OA, we checked the differential expression of the key genes using the scRNA-seq dataset and the bulk RNA-seq dataset. Since there were no normal samples in the scRNA-seq dataset, we used the Scissor algorithm (Sun et al. 2022) to identify cells highly correlated with diseased and healthy phenotype in individual single cells using bulk RNA-seq data and phenotypic information, and subsequently calculated the differential expression of key genes in the healthy and diseased cell groups. For the bulk RNA-seq datasets GSE46750 (Inflammatory cell samples from the synovium in 12 OA cases and cell samples from the normal synovium area in 12 OA cases) and GSE89408 (Synovial tissue samples from 22 OA cases and 28 healthy synovial tissue samples), the expression of key genes between the healthy and diseased samples was calculated after removing batch effects.

### Networks based drugs recommendation

To predict effective drugs, we used the SAveRUNNER algorithm, a novel network-based drug repurposing tool (Paci et al. 2022), which quantifies the proximity between drug targets and disease modules or associated proteins in the human interactome (i.e., the cellular network of all physical molecules interacting with each other) by means of a network-based similarity measure. The SAveRUNNER source code is freely available as R-code at the website (https://github.com/giuliafiscon/SAveRUNNER.git), and its required input files are a list of key gene names and a list of drug targets downloaded from the DrugBank database. The key genes we acquire will constitute disease modules, and SAveRUNNER predicts drug-disease associations through a network-based computational approach that calculates the proximity between drug targets and disease modules or associated proteins.

### Mendelian randomization (MR) analysis of candidate drug target genes expression and OA

Two sample MR analysis was used to assess the potential causal association between the expression of drug targets and OA by using gene expression quantitative trait loci (eQTL) (Sample 1) (Ngwa et al. 2022) of drug targets and genome-wide association studies of OA (Sample 2). The inverse variance weighted (IVW) method was used to calculate the relationship between drugs targets and OA. IVW has the highest frequency of application and detection efficiency among various MR methods. The target information of drugs and drug actions (inhibitor, agonist or antagonist) was obtained from Drugbank, and the significant cis-eQTLs (*P*-value < 5 × 10^–8^) of drug targets as instrumental variables were obtained from the Genotype-Tissue Expression (GTEx) database. We firstly selected significant eQTLs from fibroblasts and if there were no significant eQTLs from fibroblasts, the eQTLs from most significant tissues were selected. Drug screening was based on the criterion that at least one target was detected with significant results in fibroblasts. The GWAS data for OA were obtained from the IEU OpenGWAS projeat (https://gwas.mrcieu.ac.uk/), and nine OA GWAS datasets from recent years were selected. MR was performed using the R package “TwoSampleMR”, and sensitivity analysis of MR was performed to rule out the presence of horizontal pleiotropy, and a Cochran's Q to test for heterogeneity. Bonferroni's method was used to determine the significance threshold for multiple hypothesis testing.

### OA model in zebrafish induced by TCC

The zebrafish OA model for this experiment was established by culturing zebrafish in the living environment of TCC (Zhang et al. 2023), a widely used environmental endocrine disrupting chemical (Iacopetta et al. 2021). Exposure to TCC causes OA within the anal fin of zebrafish, with a shortened mean joint distance in TCC-exposed zebrafish compared to healthy zebrafish. This further leads to reduction of cartilage tissue and narrowing of the intra-articular space, which are typical features of the pathogenesis of OA. We first chose the 1–3 μM gradient of TCC adopted by the reference (Zhang et al. 2023), but the modeling effect of 1 μM TCC was not obvious, and most of the fish died after three weeks of incubation with 3 μM TCC, so we chose 2 μM TCC as the standard of drug concentration for constructing the OA model.

### The effect of candidate drugs on the joint space of zebrafish OA model

Zebrafish matured in culture for about three months were selected for drug experiments, and the drugs were administered in groups according to the criteria shown in supplementary Table 1 for three weeks of culture, with weekly water changes, and all groups of drugs were dissolved in a mixture of 0.025% DMSO in a volume of 1 L. The number of zebrafish in each group was 20 (n = 20). The positive control drug was chondroitin, which has anti-inflammatory activity with clinical benefit in OA by providing resistance to compression, maintaining structural integrity, homeostasis, slowing down catabolism and alleviating pain from muscle soreness (Singh et al. 2015, Bishnoi et al. 2016). Before determining the final concentration, we first conducted a preliminary experiment on zebrafish larvae. The survival rates were recorded after 72 h of treatment with different concentrations. The concentration with the highest survival rate was ultimately selected. Zebrafish incubated with the drug for three weeks were subjected to double staining of Alcian blue-alizarin red and joint clearance was measured at the hip-fin joints. The staining of zebrafish at the anal fin joints was observed under a body microscope. The images were analyzed by imageJ, an image processing analysis software (Jensen 2013), to quantify the joint gap at the anal fin joints by means of standard calipers to reflect the degree of damage at the anal fin joints.

### Reverse transcription quantitative polymerase chain reaction (RT-qPCR) to detect the expression candidate genes

To validate the expression of the key genes and drug target genes, the tissues at the anal fin from three weeks drug-treated zebrafish, three from each drug culture condition, were used for the RT-qPCR experiment. For the five key genes, MMP3 and ICAM1 genes do not have homologous genes in zebrafish and were excluded in the RT-qPCR experiment. The homologous gene of BIRC3 in zebrafish is BIRC2, the homologous gene of CXCL8 in zebrafish is CXCL8A, and the homologous gene of CCL20 in zebrafish is CCL20B. HMGCRA is the target gene for the drug Pitavastatin, and TUBB1 is the target gene for Cabazitaxel. The one-way ANOVA analysis was performed to analyze the RT-qPCR quantification results across groups, and the data were visualized using GraphPad Prism software. The primers used in RT-qPCR are shown in Supplementary Table 2.

## Results

### Cell annotation of scRNA-seq data

**Fig. 2:**
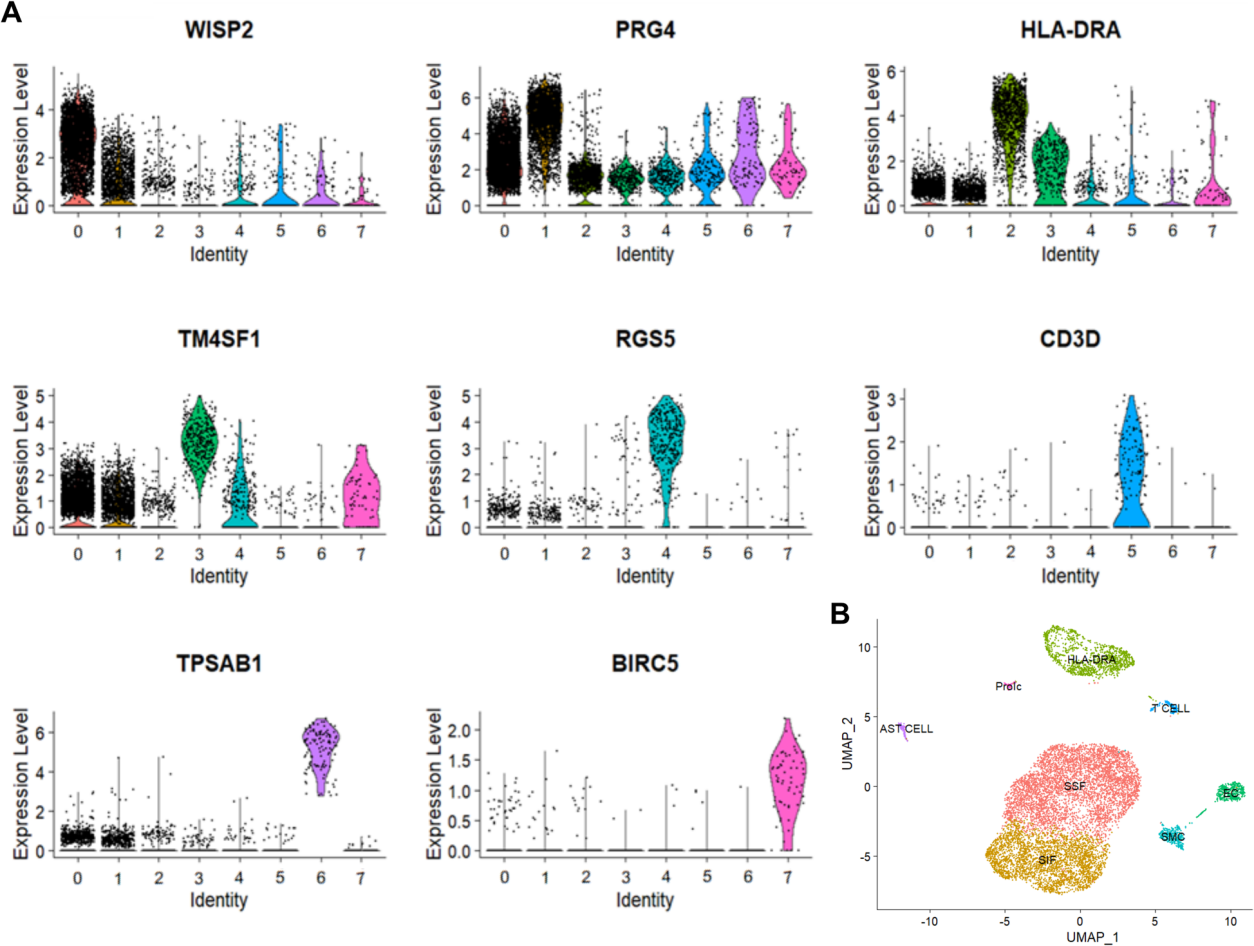
Cell annotation results. **A**, Marker gene annotated cell clusters. WISP2 is a marker gene for SSF, PRG4 is a marker gene for SIF, HLA-DRA is a marker gene for HLA-DRA + cells, TM4SF1 is a marker gene for ECs, RGS5 is a marker gene for SMCs, CD3D is a marker gene for T cells, TPSAB1 is a marker gene for mast cells, and BIRC5 is a marker gene for ProICs. **B**, Cell annotation umap plot

The single-cell dataset GSE152805 consisted of 10,345 cells, which were divided into 8 clusters. These clusters were annotated with synovial subintimal fibroblasts (SSF), synovial intimal fibroblasts (SIF), HLA-DRA^+^ cells (including immunomodulatory (IR-MΦ) and inflammatory macrophages (I-MΦ), dendritic cells (DC), activated proinflammatory cells (IFIB), B cells, T cells, proliferating immune cells (ProIC), smooth muscle cells (SMC), mast cells, and endothelial cells (EC). WISP2 is a marker gene for SSF, PRG4 is a marker gene for SIF, HLA-DRA is a marker gene for HLA-DRA + cells, TM4SF1 is a marker gene for ECs, RGS5 is a marker gene for SMCs, CD3D is a marker gene for T cells, TPSAB1 is a marker gene for mast cells, and BIRC5 is a marker gene for ProICs (Fig. 2A). The UMAP plot displays the distribution of cell types (Fig. 2B).

### Grouping of high/low inflammatory cells

**Fig. 3:**
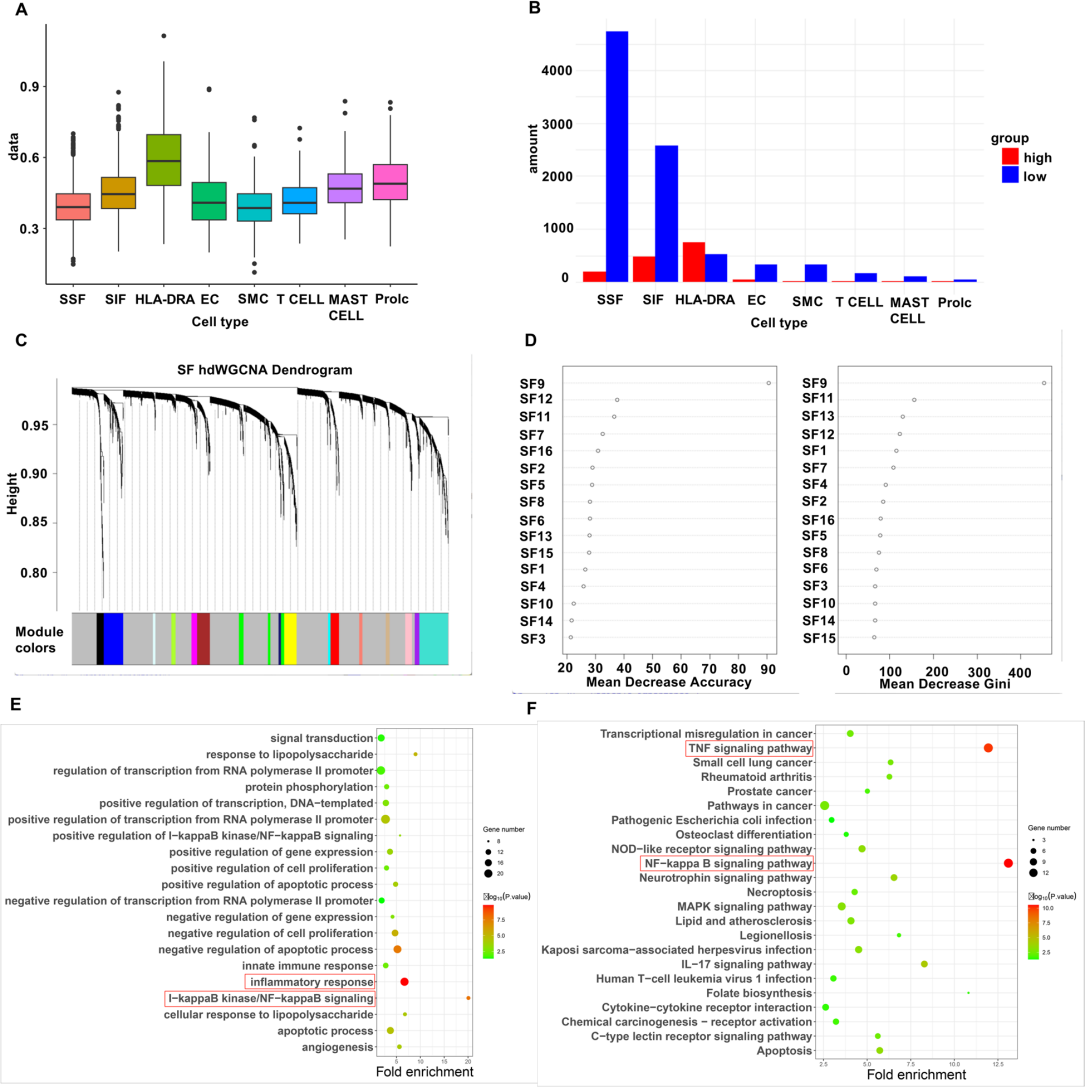
Screening of key gene modules. **A**, Cell cluster ssGSEA inflammatory gene score. X axis indicates cell type, Y axis indicates the average score. **B**, Number of high inflammatory/low inflammatory cells in different cell types. **C**, hdWGCNA module clustering results. **D**, Random forest results showed Gini coefficients of each module for screening of key gene modules. **E**–**F**, GO and KEGG enrichment analyses. Dot size in the graph indicates the number of genes enriched. Size indicates how many genes were enriched. Color change indicates -log10(*P-value*), X-axis indicates the degree of enrichment

Each cell was scored for inflammation by ssGSEA, and then the cells were categorized into high-inflammatory and low-inflammatory groups based on the median score. In Figs. 3A, we got 4740 low inflammatory cells and 201 high inflammatory cells in SSF and 2576 low inflammatory cells and 478 high inflammatory cells in SIF. Figure 3B shows the scoring of each cell cluster. We found that HLA-DRA^+^ cells have the highest inflammation scores, since some of these cells like B cells, inflammatory macrophages and pre-activated inflammatory cells are strongly associated with inflammation.

### Identification of key modules and genes associated with OA

**Fig. 4:**
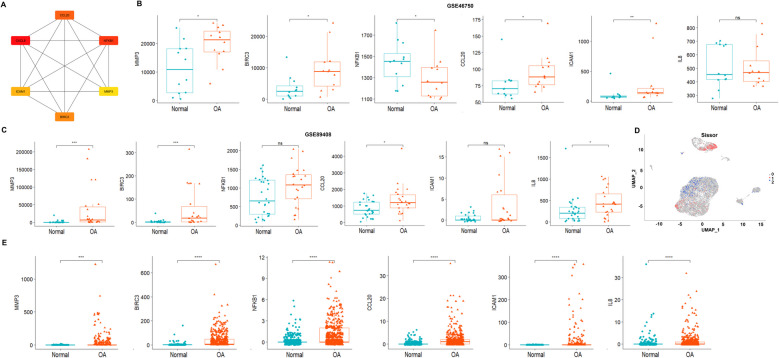
Identification of key genes. **A**, PPI key gene module. **B**, Validation of gene expression in bulk dataset GSE46750. **C**, Validation of gene expression in bulk dataset GSE89408. **D**, Scissor algorithm results, 742 red cell taxa1 for calculated diseased cell subpopulations and 583 blue cell taxa2 for calculated healthy disease subpopulations. **E**, Validation of gene expression in single-cell dataset GSE152805. Statistical significance (ns *P*-value > 0.05, * P-value < 0.05, ** P-value < 0.01, *** P-value < 0.001, **** P-value < 0.0001)

Seventeen modules other than the gray module were obtained by hdWGCNA analysis (Fig. 3C). Random Forest algorithm showed that the SF9 module had a Gini coefficient much higher than the other modules, and thus was considered as the most important key disease module (Fig. 3D). GO enrichment analysis on 163 genes of the SF9 module showed that the module genes were significantly enriched in inflammation and angiogenesis (Fig. 3E). KEGG analysis showed that the module genes were significantly enriched in signaling pathways such as TNF signaling pathway and NF-KB signaling pathway (Fig. 3F). We constructed the PPI network by ranking the top 20 genes in SF9 module in terms of connectivity. Then the MCC algorithm constructed a key sub-network composed of 6 genes (CXCL8, CCL20, MMP3, BIRC3, ICAM1 and NFKB1) (Fig. 4A).

### Validation of OA related key genes

To validate whether the six genes by PPI network analysis are related to osteoarthritis, we checked the differential expression of the six genes. In the Bulk RNA-seq data, 5 key genes (CXCL8, CCL20, MMP3, BIRC3, ICAM1 and NFKB1) showed significantly higher expression in the OA group than in the normal group. However, NFKB1 showed inconsistent expression between OA and control group in the two Bulk RNA-seq data, this gene was removed from the following analysis (Fig. 4B-C). In the single-cell dataset, the scissor algorithm showed that there were a total of 742 cells as the diseased group, and 583 cells as the healthy group (Fig. 4D), and the differential expression analysis showed that the expression of five key genes in the disease group were significantly higher than those of the healthy group (Fig. 4E).

### Candidate drugs for OA treatment

Using SAveRUNNER algorithm, we got a total of 42 drugs related to osteoarthritis based on the ranking of adjusted_similarity value (Table 1). Some of these drugs were known anti-osteoarthritis drugs, indicating the reliability of our drug repositioning method. For example, Glucosamine was a known OA preventive and therapeutic drug, which is necessary for the synthesis of proteoglycan in the cartilage matrix of the human joints and can help repair and maintain cartilage and stimulate the growth of chondrocytes. Adalimumab was an anti-tumor necrosis factor (TNF) drug used to treat inflammatory joint conditions such as rheumatoid arthritis. Colchicine was usually used to treat gout, but sometimes used in the treatment of arthritis.

**Table 1:** Candidate drugs recommended by SAveRUNNER algorithm

Drug	Proximity	*P*-value	Similarity	Adjusted_similarity
adalimumab	1	5.46E-07	0.8	9.99E-01
aldesleukin	1.3	3.36E-08	0.7	9.98E-01
alteplase	1.5	1.04E-05	0.7	9.97E-01
amrinone	1.7	3.59E-02	0.6	9.92E-01
anistreplase	1.5	1.09E-04	0.7	9.97E-01
auranofin	1.5	6.74E-03	0.7	9.97E-01
berberine	1	9.41E-10	0.8	9.99E-01
Cabazitaxel	1.5	8.82E-03	0.7	9.97E-01
certolizumab pegol	1	1.37E-04	0.8	9.99E-01
colchicine	1	3.43E-02	0.8	9.99E-01
denileukin diftitox	1.3	2.68E-07	0.7	9.98E-01
denosumab	1	4.84E-05	0.8	9.99E-01
dipyrithione	1.5	2.19E-02	0.7	9.97E-01
drotrecogin alfa	1.9	9.53E-03	0.6	9.86E-01
glucosamine	1.5	9.76E-05	0.7	9.97E-01
golimumab	1	4.79E-07	0.8	9.99E-01
human calcitonin	1	6.83E-03	0.8	9.99E-01
hyaluronic acid	1.8	3.73E-02	0.6	9.90E-01
infliximab	1	3.42E-06	0.8	9.99E-01
ketoprofen	1.6	1.26E-02	0.6	9.95E-01
lifitegrast	1	3.4E-04	0.8	9.99E-01
lovastatin	1.6	3.65E-02	0.6	9.95E-01
mecasermin	1.7	1.05E-02	0.6	9.92E-01
mifamurtide	1.5	6.63E-06	0.7	9.97E-01
minocycline	1.5	1.52E-05	0.6	9.96E-01
Pitavastatin	1.5	8.10E-03	0.7	9.97E-01
podofilox	1.3	1.30E-02	0.7	9.98E-01
pomalidomide	1.6	1.31E-03	0.6	9.95E-01
reserpine	1.5	4.55E-03	0.7	9.97E-01
reteplase	1.5	8.19E-07	0.7	9.97E-01
rosuvastatin	1.5	1.38E-02	0.7	9.97E-01
sargramostim	1.8	2.55E-02	0.6	9.91E-01
streptokinase	1.5	2.03E-03	0.7	9.97E-01
sucralfate	1.8	2.72E-02	0.6	9.91E-01
tasonermin	1.5	3.36E-02	0.7	9.97E-01
tenecteplase	1.8	2.83E-02	0.6	9.90E-01
tranexamic acid	1	7.67E-09	0.8	9.99E-01
triflusal	1.5	2.25E-02	0.7	9.97E-01
vinblastine	1.6	3.22E-02	0.6	9.95E-01
vincristine	1	3.01E-04	0.8	9.99E-01
vinorelbine	1	3.83E-02	0.8	9.99E-01
xanthinol	1.8	4.81E-02	0.6	9.90E-01

### MR analysis to estimate the association of drug target genes expression with OA

To estimate the potential effect of drug candidates on OA, we performed MR analysis to determine the association between the expression of 42 drug targets and OA. Among the 42 drugs, gene expressions of 10 drug targets were detected to be associated with OA (*P* < 0.05/(8*42) (Supplementary Table 3). After considering various factors like drug experiment ability, reproducibility of different drug targets, and whether significant results were detected in fibroblasts, two drugs (Cabazitaxel and Pitavastatin) would draw our interest for further investigation.

Cabazitaxel has two targets, TUBA4A and TUBB1. TUBA4A had no significant cis-eQTLs, and TUBB1 was tested using cis-eQTLs in fibroblast and thyroid tissues. IVW results showed the low expression of Cabazitaxel targets TUBB1, which are inhibited by Cabazitaxel, was associated with increased risk of OA ($\upbeta$ = −0.08, *P*-value = 4.56 E-04 in ebi-a-GCST007091, Table 2, Supplementary Fig. 1). Therefore, Cabazitaxel can increase the risk of OA. We observed that there was no significant heterogeneity among the instruments (*P*-value = 0.94) and horizontal pleiotropic effects (*P*-value = 0.53). The leave-one-out test showed that removing any SNP would not have a fundamental impact on the outcome, suggesting that the MR results were robust (Supplementary Fig. 1).

**Table 2:** MR analysis of Cabazitaxel targets and OA

Data	Target	Method	β	*P*-value	Organization
ebi-a-GCST007091	TUBB1	IVW	−7.81 E-02	4.56 E-04	Fibroblasts
ukb-b-14486	TUBB1	IVW	−3.14 E-03	3.16 E-02	Fibroblasts
ukb-a-106	TUBB1	IVW	−3.12 E-03	3.95 E-02	Fibroblasts
ebi-a-GCST007092	TUBB1	IVW	−2.83 E-02	3.93 E-02	Thyroid
ebi-a-GCST007091	TUBB1	IVW	−7.38 E-02	4.39 E-04	Thyroid
ebi-a-GCST005811	TUBB1	IVW	−5.06 E-02	4.63 E-02	Thyroid
ukb-b-14486	TUBB1	IVW	−4.04 E-03	1.23 E-04	Thyroid

Pitavastatin, is used to lower lipid levels and reduce the risk of cardiovascular disease, including myocardial infarction and stroke (Leake 1978). Pitavastatin has two targets HMGCR and ITGAL. IVW results showed the low expression of Pitavastatin targets HMGCR and ITGAL, which are inhibited by Pitavastatin, was associated with reduced risk of OA (for HMGCR $\upbeta$ = 0.13, *P*-value = 2.67E-06, for ITGAL $\upbeta$ = 0.08, *P*-value = 6.57E-08, Table 3). Therefore, Pitavastatin can reduce the risk of OA. We observed that there was no significant heterogeneity among the instruments (*P*-value = 0.97) and horizontal pleiotropic effects (*P*-value = 0.96). The leave-one-out test showed that removing any SNP would not have a fundamental impact on the outcome, suggesting that the MR results were robust (Supplementary Fig. 1).

**Table 3:** MR analysis of Pitavastatin targets and OA

Data	Target	Method	β	*P*-value	Organization
ebi-a-GCST007092	HMGCR	IVW	6.73 E-02	4.01 E-03	Muscle-Skeletal
ebi-a-GCST007090	HMGCR	IVW	1.34 E-01	2.67 E-06	Muscle-Skeletal
ebi-a-GCST005813	HMGCR	IVW	2.23 E-01	2.29 E-03	Muscle-Skeletal
ebi-a-GCST007092	ITGAL	IVW	4.28 E-02	2.45 E-04	Fibroblasta
ebi-a-GCST007090	ITGAL	IVW	7.62 E-02	6.57 E-08	Fibroblasta

### RT-qPCR validation of OA-related key genes and drug target genes

**Fig. 5:**
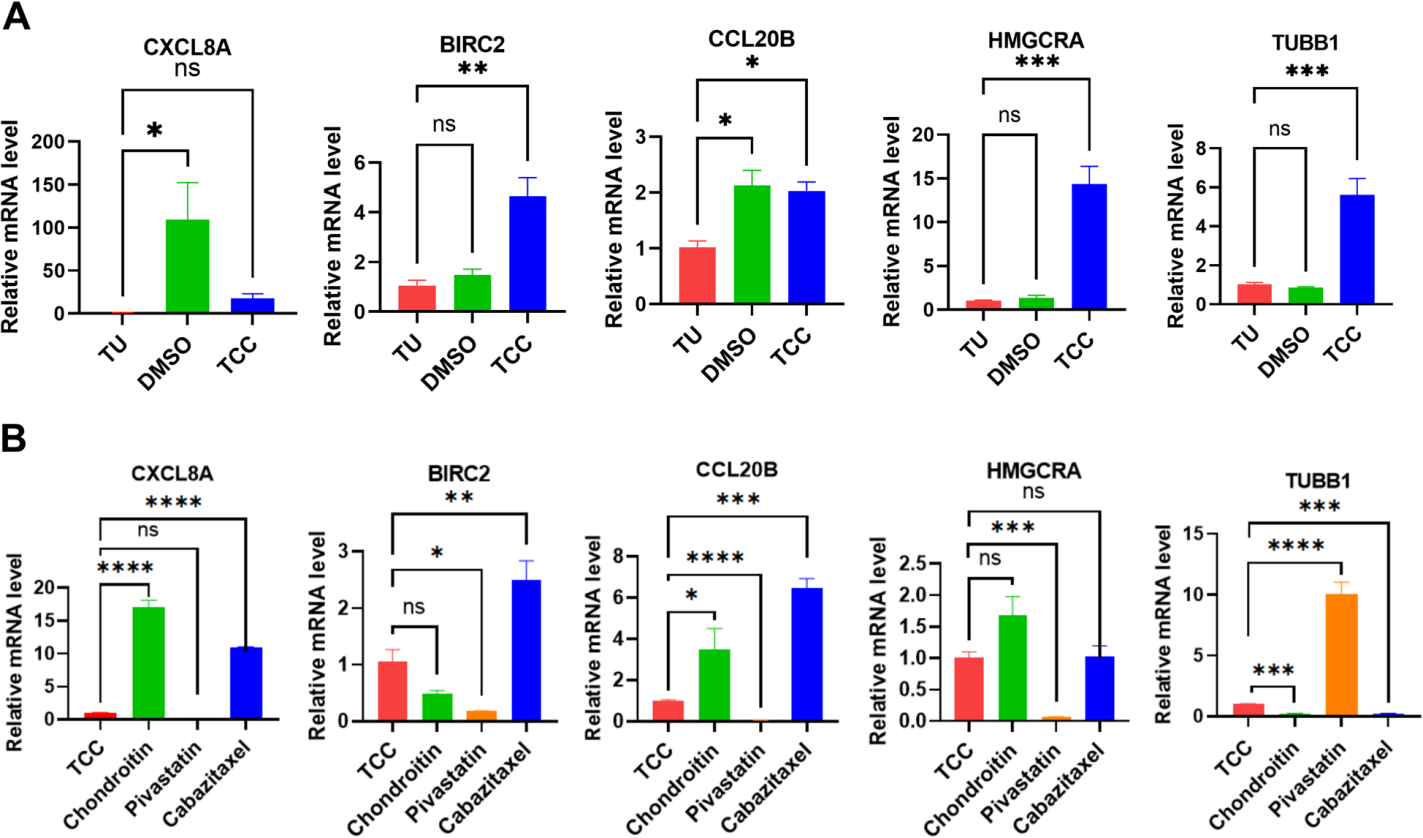
Results of RT-qPCR experiments. **A**, RT-qPCR results of genes in wild type, DMSO, and TCC groups. **B**, RT-qPCR results of different drug treatment groups. Statistical significance (ns *P*-value > 0.05, * P-value < 0.05, ** P-value < 0.01, *** P-value < 0.001, **** P-value < 0.0001)

Figure 5A shows that the transcript levels of OA-related key genes (BIRC2, CXCL8 and CCL20B) were significantly higher in the TCC group compared to the wild type group, indicating that the key genes identified by the present study contributed to the pathological mechanism of OA. The transcript levels of the three key genes (BIRC2, CXCL8 and CCL20B) were significantly lower in the Pitavastatin group than in the TCC group, and their expression was significantly higher in the Cabazitaxel group than in the TCC group. The transcript levels of HMGCRA, the drug target of Pitavastatin, were significantly lower than those of the TCC group, and the expression of TUBB1, the drug target of Cabazitaxel, was significantly lower than that of the TCC group, suggesting that the drug successfully acted on the targets (Fig. 5B).

### Effect of candidate drugs on joint gap in zebrafish

To test the effect of drug candidates Cabazitaxel and Pitavastatin on TCC-induced OA model, zebrafish cultured for three weeks under different conditions were double stained with Alcian blue-alizarin red in order to observe the changes of the joint space at the anal fin of the zebrafish. In the pre-experiment, we found that 2μM TCC (Supplementary Fig. 3 A), 0.1μg/mL Cabazitaxel (Supplementary Fig. 3B), and 0.5μg/mL Pitavastatin (There was no significant difference between the 0.1μg/mL and 0.5μg/mL groups) (Supplementary Fig. 3 C) showed the best survival rate of the zebrafish larvae, so the drug concentrations were set to 2μM TCC, 0.1μg/mL Cabazitaxel, 0.5μg/mL Pitavastatin.

**Fig. 6:**
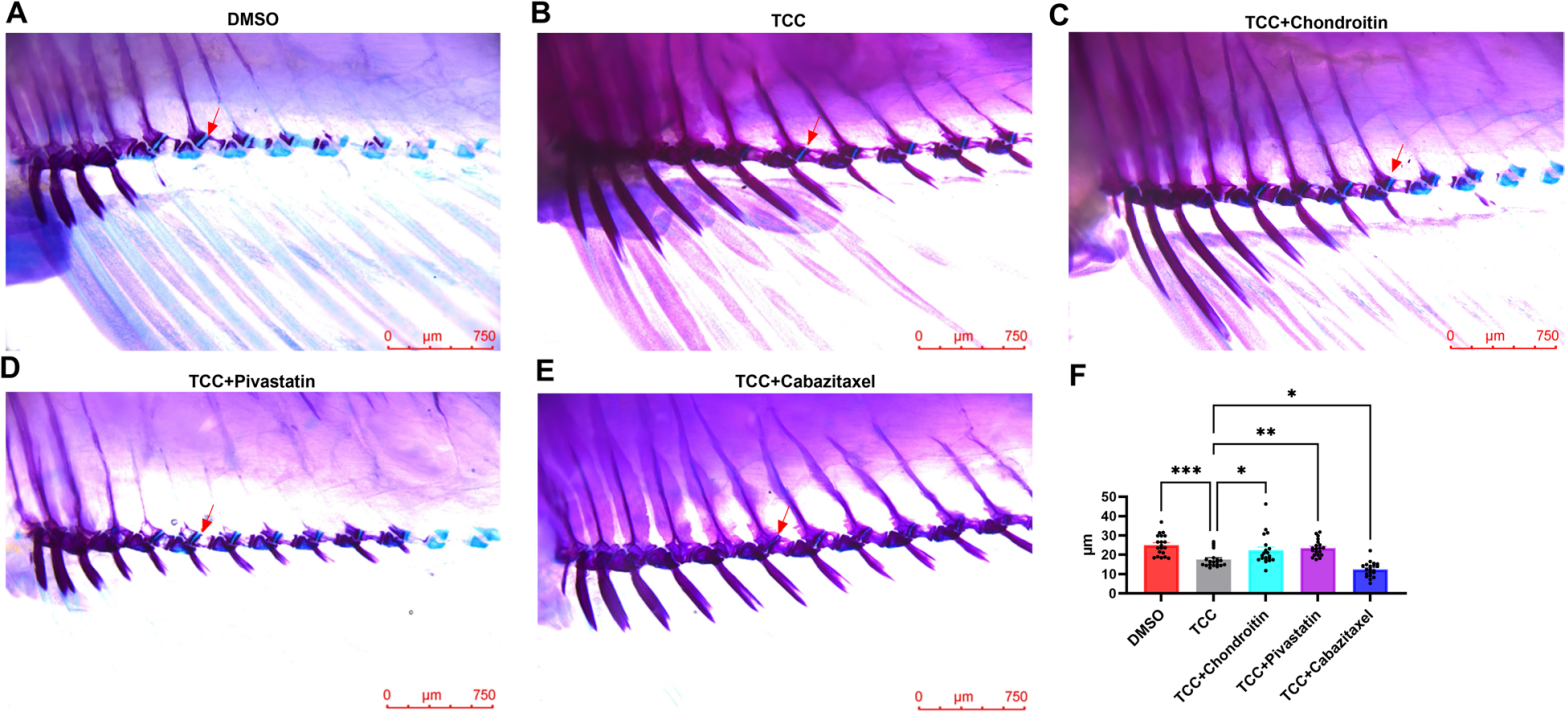
Effect of different drug treatments on the joint space. **A**, 0.025% DMSO treated group. **B**, 0.025% DMSO + 2μM TCC treated group. **C**, 0.025% DMSO + 2μM TCC + 10μg/mL chondroitin treated group. **D**, 0.025% DMSO + 2μM TCC + 0.1μg/mL Cabazitaxel Treatment group. **E**, 0.025% DMSO + 2μM TCC + 0.5μg/mL Pitavastatin Treatment group. **F**, Mean joint space results of different drug treatment groups. Statistical significance (* *P*-value < 0.05, ** *P*-value < 0.01, *** *P*-value < 0.001)

The experimental results showed that compared with the TCC group, the joint gap in the Pitavastatin group was significantly increased (17.54 ± 4.22μm *vs* 23.50 ± 4.21μm, *P*-value = 0.0051), indicating that Pitavastatin can increase the joint gap in the OA model, and its effect is similar to that of chondroitin (joint gap 22.28 ± 7.82μm), which has therapeutic effect on OA, while the joint gap in the Cabazitaxel group was significantly decreased than that in the TCC group (17.54 ± 4.22μm *vs*12.41 ± 3.82μm, *P*-value = 0.0230), indicating that the Cabazitaxel increases the risk of OA (Fig. 6).

## Discussion

The most representative symptom of osteoarthritis is inflammation of the synovium. Synovium fibroblasts have an important relationship with the onset and progression of OA, and promote neuronal growth associated with OA pain (Conaghan et al. 2019). The present study used a set of inflammatory genes to score all the cells and classify them into high-inflammation and low-inflammation groups by single-sample immuno-infiltration, then used a RF algorithm to filter out the inflammation-related key module in the high-dimensional co-expression network analysis. PPI network identified five inflammation-related key genes (CXCL8, CCL20, MMP3, BIRC3 and ICAM1). Bioinformatics methods and animal experiments suggested that Pitavastatin has therapeutic effects on osteoarthritis, while Cabazitaxel increases the risk of osteoarthritis.

In this study, five key genes were identified, among which the protein encoded by CXCL8 gene is a member of the CXC chemokine family and is the main mediator of inflammatory response. The encoded protein is often referred to as interleukin-8 (IL-8) and can be secreted by fibroblasts. CXCL8 and the receptor contribute to the elimination of pathogens, but may also significantly promote disease-related processes, including tissue damage, fibrosis, angiogenesis, and tumorigenesis (Russo et al. 2014). CCL20 is an antimicrobial gene belonging to a subfamily of small cytokine CC genes. Cytokines are a family of secreted proteins involved in the recruitment of pro-inflammatory IL17-producing helper T cells (Th17) and regulatory T cells (Treg) to the site of inflammation. CCL20 induces gene expression of IL-6 and COX-2 in FLS, and upregulates the release of IL-6 and MMP3 (Alaaeddine et al. 2011). Proteins of the matrix metalloproteinase (MMP) family are involved in the breakdown of the extracellular matrix during normal physiological processes as well as disease processes (e.g., arthritis). MMP3 encodes an enzyme that degrades fibronectin, laminin, collagen III, IV, IX, and X, as well as cartilage proteoglycans, and is involved in chondrocyte apoptosis and cartilage destruction, playing a key role in OA (Shi et al. 2016). The BIRC3 gene encodes a member of the inhibitors of apoptosis protein (IAP) family, which is a multifunctional protein that regulates not only caspases and apoptosis, but also inflammatory signaling and immunity, mitotic kinase signaling, and cell proliferation. BIRC3 is a direct target of ATF6α, and model mice with ATF6α deficiency exhibit reduced arthritis progression, resulting in significant reductions in clinical and pro-inflammatory markers in the joints (Ge et al. 2022). The intercellular adhesion molecule-1 (ICAM-1) gene encodes a cell surface glycoprotein that is normally expressed on endothelial cells and immune system cells. ICAM-1 is a key regulator of monocyte recruitment to synovial tissue, and high levels of ICAM-1 expression are found in the synovium of patients with OA (Law et al. 2020).

Our study suggested that Pitavastatin has therapeutic effects on OA. In addition to lowering blood lipids and reducing the risk of cardiovascular disease, Pitavastatin improves endothelial function, reduces inflammation, and has beneficial extrahepatic effects on the immune system, the central nervous system, and the skeleton (Leake 1978). We hypothesize that the drug’s mechanism of action in response to OA is primarily through attenuating inflammatory responses. Statins have a variety of anti-inflammatory effects, including decreasing T-cell recruitment and activation, decreasing the expression of inflammatory cytokines and chemokine expression, attenuating the expression of cluster of differentiation 40 (CD40, a member of the TNF family) in T cells and lymphocytes, and inhibiting smooth muscle cell proliferation (Sadowitz et al. 2010). By inhibiting mevalonate synthesis, statins can reduce leukocyte migration through a mechanism involving monocyte chemotactic protein-1 (MCP-1), which further modulates the vascular inflammatory response (Romano et al. 2000). Pitavastatin has also been shown to have an effect on inflammation and immune regulation, which may be due in part to a dose-dependent inhibition of monocyte proliferation through downregulation of the chemokine receptors CCR2 and CCR5 (Fujino et al. 2006), an effect mediated by inhibition of reactive oxygen species production induced by Rac-1, a rat sarcoma (Ras)-associated C3 botulinum toxin substrate 1 (Kaneyuki et al. 2007).

**Fig. 7:**
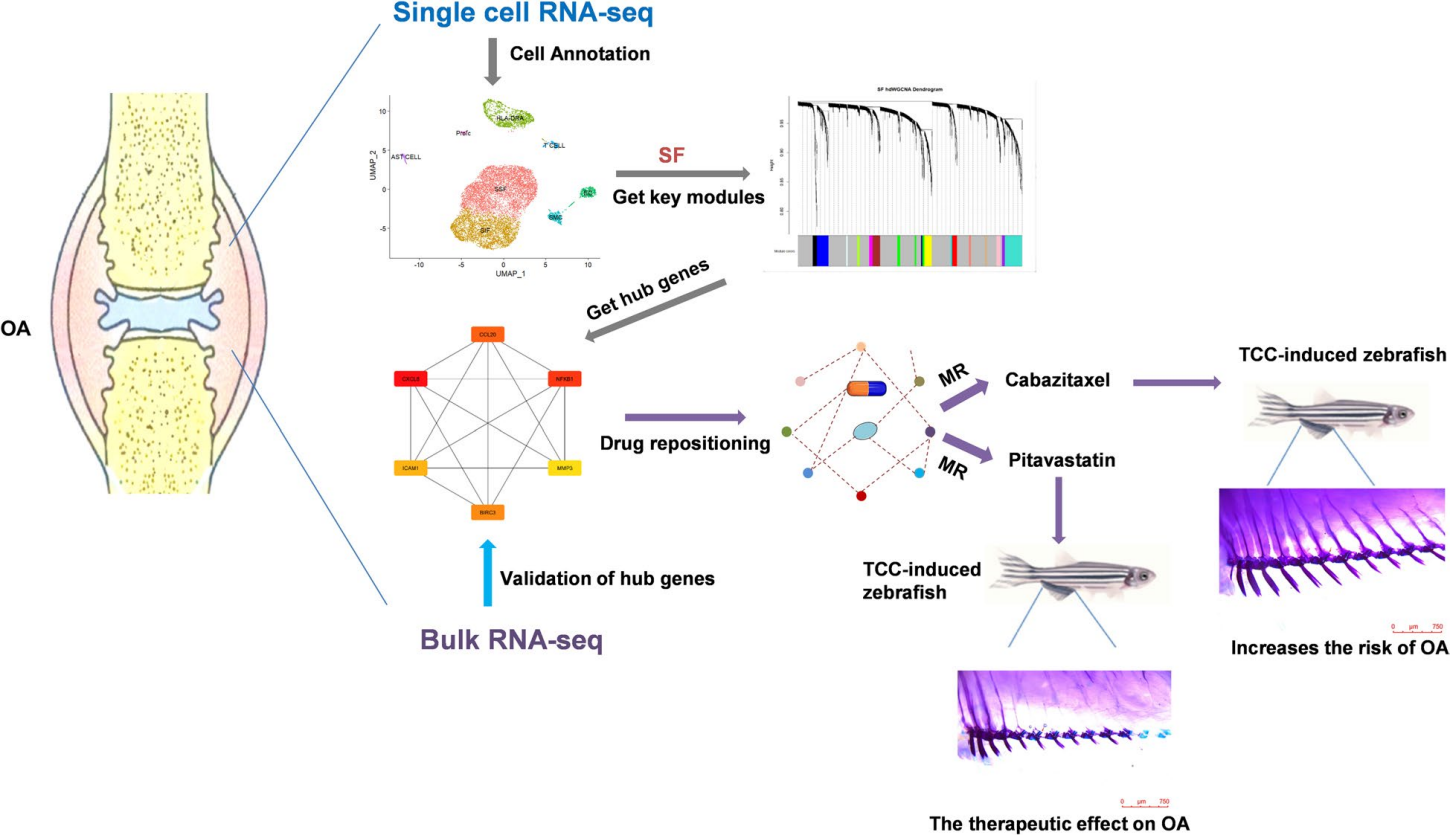
Schematic overview of this study. After performing single-cell sequencing on OA synovial tissue and annotating the cells, we used hdWGCNA analysis to identify key modules related to SF and got hub genes, which were then validated in bulk data. Based on network proximity analysis for drug repositioning, we screened candidate drugs by using MR analysis and validated their effects in a zebrafish OA model. This led to the finding that Pitavastatin has a therapeutic effect on OA, while Cabazitaxel increases the risk of OA

Furthermore, our study suggested that Cabazitaxel increases the risk of OA. Cabazitaxel has a therapeutic role in advanced prostate cancer through microtubule kinetic inhibition, which blocks tumor cell proliferation and mitosis, especially in patients who have failed docetaxel therapy (Nightingale and Ryu 2012). During mitosis, microtubules extend towards the mitotic spindle to allow segregation and distribution of chromosomes during cell division (Nightingale and Ryu 2012). Cabazitaxel binds to the N-terminal amino acid of the β-microtubulin subunit and promotes microtubule polymerization while inhibiting catabolism, which leads to microtubule stabilization and prevents microtubule cytokinesis. Cabazitaxel ultimately blocks mitotic and interphase cell function and tumor proliferation (Paller and Antonarakis 2011, Villanueva et al. 2011). It is speculated that its effect on osteoarthritis is primarily due to the fact that Cabazitaxel, as a microtubule stabilizer, may affect cell growth and division. In OA, this action may affect the growth and repair of cartilage and other tissues around the joint. Figure 7 shows the schematic overview of this study.

### Limitations

MR has several limitations. First, the choice of exposure factors, such as drug target gene expression (cis-eQTL), may not fully capture the drug’s mechanism of action. Moreover, the selected genetic regions must effectively replicate the drug’s action for MR results to be practically applicable. MR may also be limited when the drug’s biological mechanism involves changes in protein function or other modifications not reflected by gene expression.

In animal models, the zebrafish model used in this study has certain limitations. Results obtained from zebrafish may not fully translate to humans, and joint gap measurement may not encompass the full physiological complexity of OA. While zebrafish were employed to validate drug effects, further studies in mammalian models, such as mice, would provide more reliable and translatable results. Additionally, this study focused on the individual effects of chondroitin and Pitavastatin; however, exploring their combined effects could open new therapeutic avenues. The exclusion of multi-targeted drugs with conflicting effects emphasizes the need for more comprehensive treatments, as OA is a multifactorial disease. Finally, further investigations into protein expression levels and downstream pathways are needed to better elucidate the mechanisms of drug action in OA.

## Conclusion

In conclusion, this study utilized bioinformatics methods based on single-cell data identified that the five key genes (CXCL8, CCL20, MMP3, BIRC3 and ICAM1) played important roles in the pathogenesis of OA and Pitavastatin has therapeutic effects on OA, while Cabazitaxel increases the risk of OA. It provides new ideas for finding effective therapeutic drugs for diseases and helps us in the precise prevention and treatment of OA.

## Supplementary Information

Supplementary Material 1: Supplementary Figure 1. MR analysis of Cabazitaxel. A, Inferred causal relationship between Cabazitaxel drug target TUBB1 and OA by different MR methods. B, Sensitivity of leave-one-out detection between Cabazitaxel drug target TUBB1 and OA

Supplementary Material 2: Supplementary Figure 2. MR analysis of Pitavastatin. A, Different MR methods for inferring the causal relationship between the Pitavastatin drug target HMGCR and OA. B, Leave-one-out sensitivity analysis between the Pitavastatin drug target HMGCR and OA

Supplementary Material 3: Supplementary Figure 3. Concentration screening of drugs based on survival rate in zebrafish larvae. A, TCC. B, Cabazitaxel. C, Pitavastatin. Statistical significance

Supplementary Material 4.

Supplementary Material 5.

## Data Availability

All the datasets could be downloaded directly from the indicated websites.

## Abbreviations

AST CELL: Mast cells
DC: Dendritic cells
DEGs: Differential expressed genes
EC: Endothelial cells
eQTL: Expression quantitative trait loci
FLS: Fibroblast-like synoviocytes
GEO: Gene Expression Omnibus
GO: Gene ontology
GTEx: Genotype-Tissue Expression project
hdWGCNA: High-dimensional Weighted Correlation Network Analysis
I-MΦ: Inflammatory macrophages
IR-MΦ: Immunoregulatory macrophages
IVW: Inverse variance weighting method
KEGG: Kyoto Encyclopedia of Genes and Genomes
MEs: Module Enrichment analysis
MR: Mendelian randomization
MSigDB: Molecular Signatures Database
OA: Osteoarthritis
PPI: Protein–protein interaction network
ProIC: Proliferative index
RF: Random forest algorithm
scRNA-seq: Single-cell RNA sequencing technology
SIF: Synovial inflammation factor
SMC: Smooth muscle cells
SSF: Synovial stromal cells
SsGSEA: Single-sample Gene Set Enrichment Analysis
STRING: Search Tool for the Retrieval of Interacting Genes/Proteins
TCC: Tricarballylic acid cycle
Treg: Regulatory T cells
UMAP: Uniform Manifold Approximation and Projection
