# Supplementary figures and images for: Identification of osteoarthritis-related genes and potential drugs based on single cell RNA-seq data
Source: Mol Med. 2025 Nov 25;32:1. doi: 10.1186/s10020-025-01379-z (PMC12763847; doi:10.1186/s10020-025-01379-z)

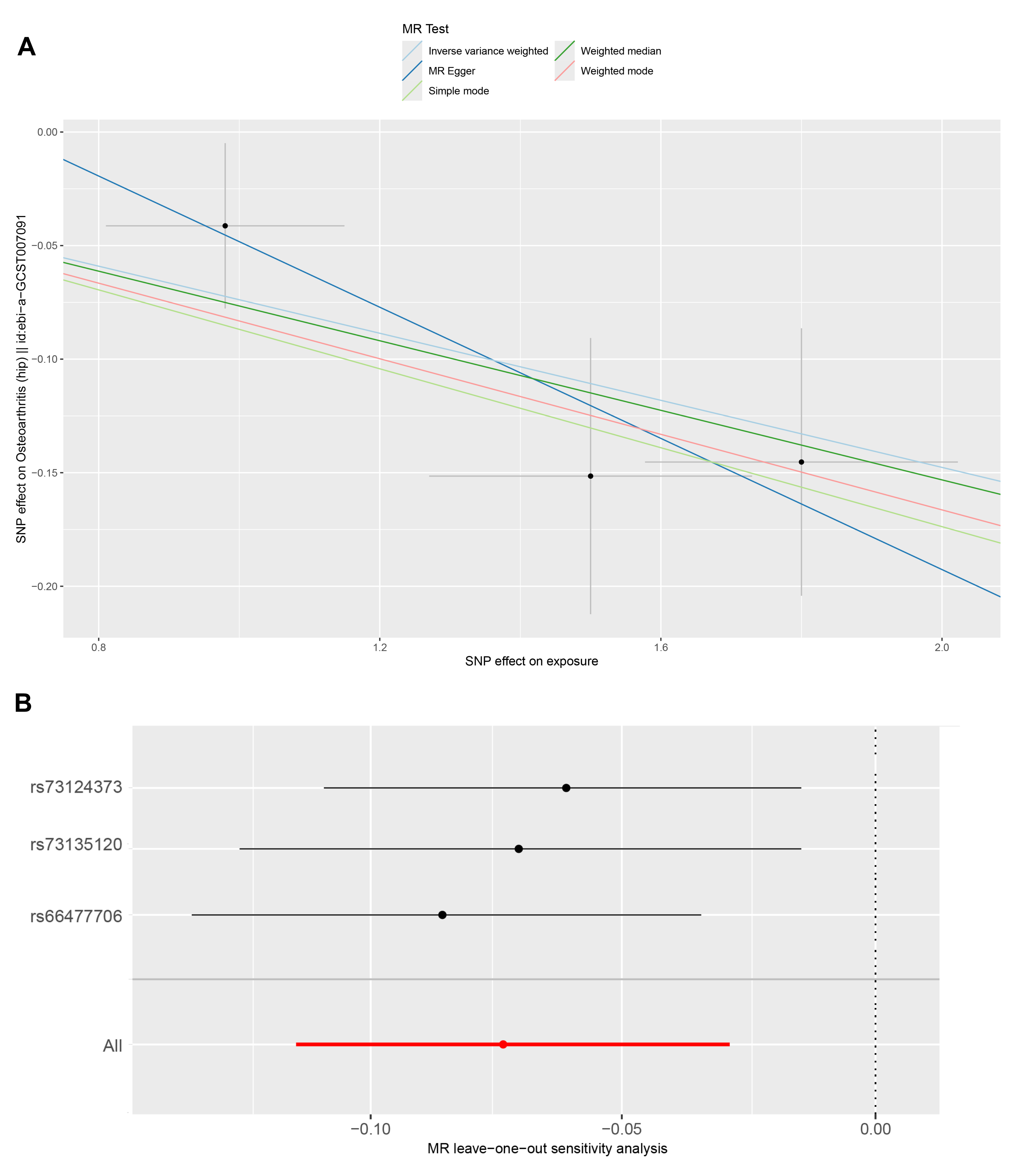

Supplement: Supplementary file 1 — Supplementary Material 1: Supplementary Figure 1. MR analysis of Cabazitaxel. A, Inferred causal relationship between Cabazitaxel drug target TUBB1 and OA by different MR methods. B, Sensitivity of leave-one-out detection between Cabazitaxel drug target TUBB1 and OA [file 10020_2025_1379_MOESM1_ESM.tif]

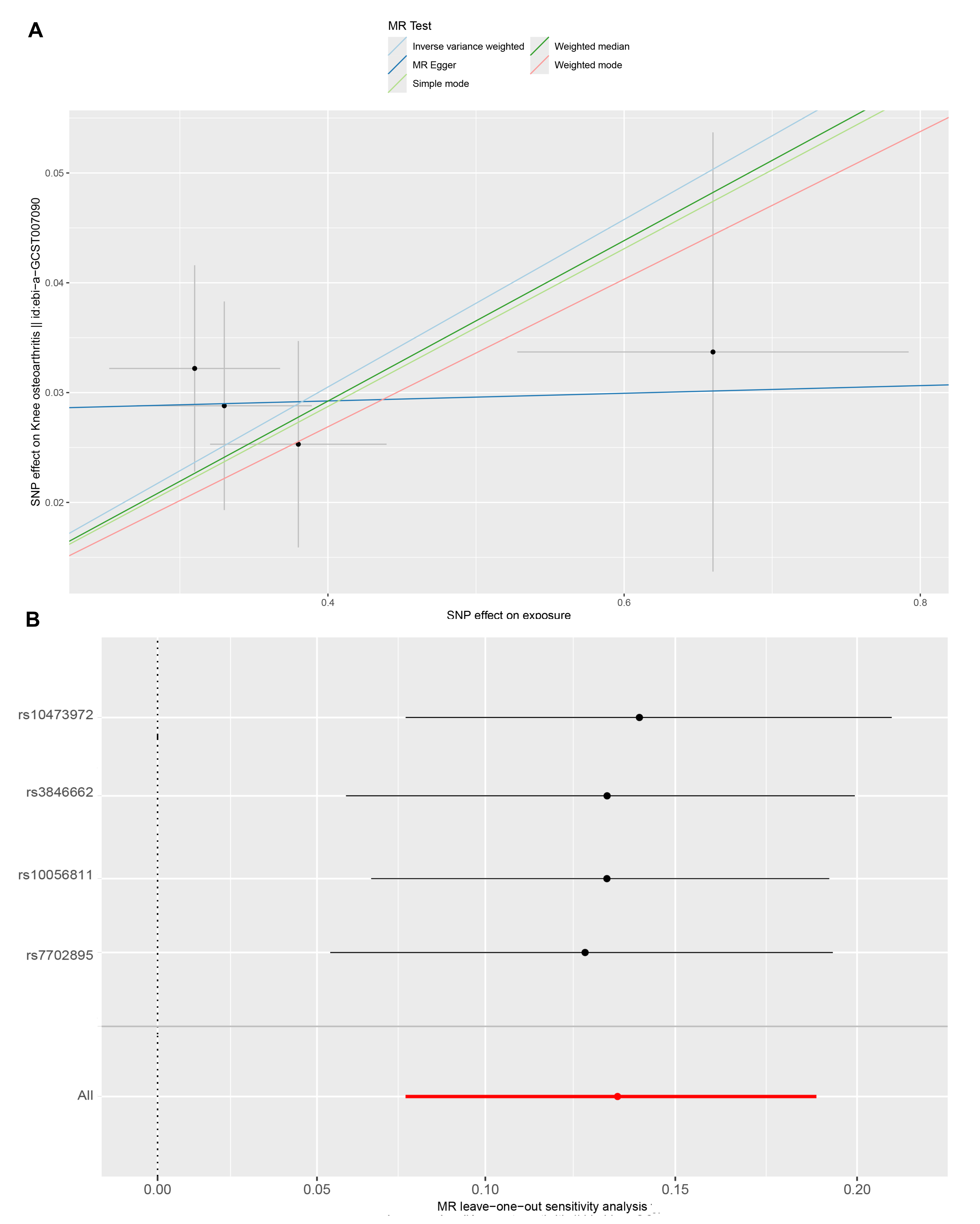

Supplement: Supplementary file 2 — Supplementary Material 2: Supplementary Figure 2. MR analysis of Pitavastatin. A, Different MR methods for inferring the causal relationship between the Pitavastatin drug target HMGCR and OA. B, Leave-one-out sensitivity analysis between the Pitavastatin drug target HMGCR and OA [file 10020_2025_1379_MOESM2_ESM.tif]

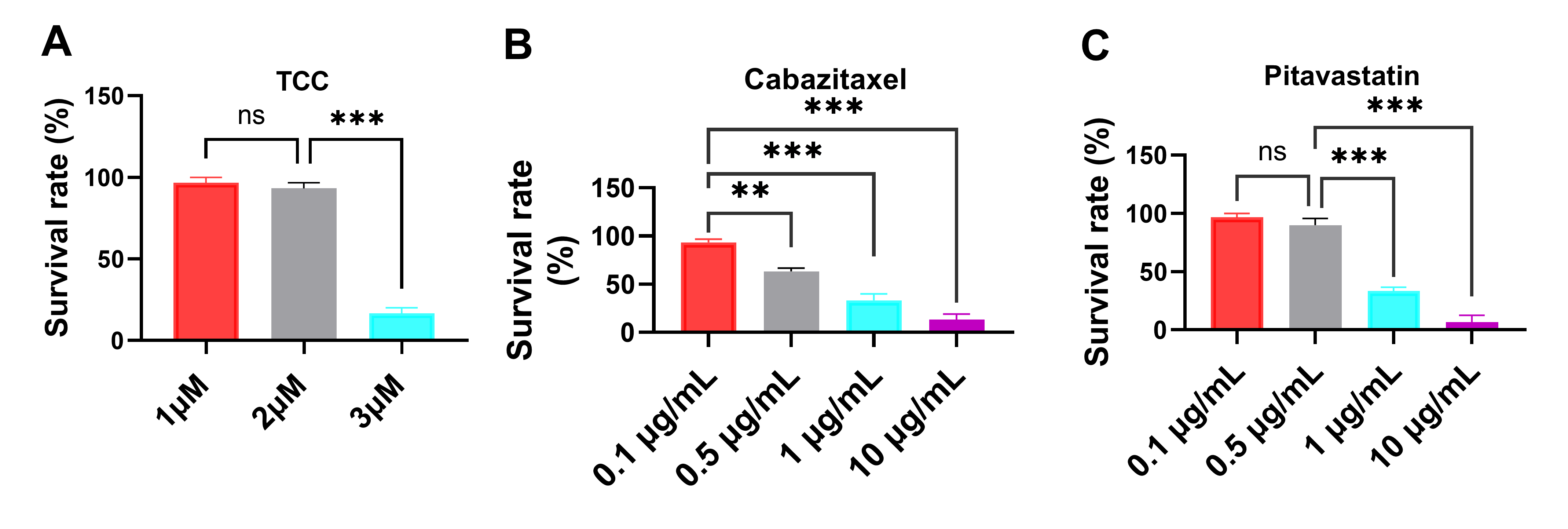

Supplement: Supplementary file 3 — Supplementary Material 3: Supplementary Figure 3. Concentration screening of drugs based on survival rate in zebrafish larvae. A, TCC. B, Cabazitaxel. C, Pitavastatin. Statistical significance [file 10020_2025_1379_MOESM3_ESM.tif]
